# Cellulose Nanofibers as a Modifier for Rheology, Curing and Mechanical Performance of Oil Well Cement

**DOI:** 10.1038/srep31654

**Published:** 2016-08-16

**Authors:** Xiuxuan Sun, Qinglin Wu, Sunyoung Lee, Yan Qing, Yiqiang Wu

**Affiliations:** 1 School of Renewable Natural Resources, Louisiana State University AgCenter, Baton Rouge, Louisiana 70803, United States; 2 Department of Forest Products, Korea National Institute of Forest Science, Seoul, 130-712, Korea; 3 College of Materials Science and Engineering, Central South University of Forestry and Technology, Changsha, 410004, China

## Abstract

The influence of nanocellulose on oil well cement (OWC) properties is not known in detail, despite recent advances in nanocellulose technology and its related composite materials. The effect of cellulose nanofibers (CNFs) on flow, hydration, morphology, and strength of OWC was investigated using a range of spectroscopic methods coupled with rheological modelling and strength analysis. The Vom-Berg model showed the best fitting result of the rheology data. The addition of CNFs increased the yield stress of OWC slurry and degree of hydration value of hydrated CNF-OWC composites. The flexural strength of hydrated OWC samples was increased by 20.7% at the CNF/OWC ratio of 0.04 wt%. Excessive addition of CNFs into OWC matrix had a detrimental effect on the mechanical properties of hydrated CNF-OWC composites. This phenomenon was attributed to the aggregation of CNFs as observed through coupled morphological and elemental analysis. This study demonstrates a sustainable reinforcing nano-material for use in cement-based formulations.

Cementing plays a crucial role in the oil well drilling operation-protecting and supporting casing, preventing fluid migration, and sealing abandoned sections of a well. Improper cementing work can cause serious consequences for oil exploration operations. For example, the Deepwater Horizon accident, which is one of the USA’s worst offshore oil disasters, brought about great environmental pollution and economic loss^1^. According to the Deepwater Horizon accident investigation report, one major cause of the accident was faulty cement work, which led to a significant financial settlement to resolve the spill lawsuits for the industry.

Among various cements, Class H oil well cement (OWC) is used by nearly 80% of oil drilling companies, which is manufactured by grinding ordinary Portland cement clinker and blending them with the other additives. OWC is mainly composed of tricalcium silicate (3CaO·SiO_2_ or “C_3_S”), dicalcium silicate (2CaO·SiO_2_ or “C_2_S”), tricalcium aluminate (3CaO·Al_2_O_3_ or “C_3_A”) and tetracalcium alumino ferrite (4CaO·Al_2_O_3_·Fe_2_O_3_ or “C_4_AF”). Gypsum (CaSO_4_·2H_2_O) is also added to control setting time and improve final cement strength. When OWC is mixed with water, a series of complex chemical reactions occur. Calcium silicate hydrate (C-S-H) gel and calcium hydroxide are two major products from the reaction of C_3_S and C_2_S with water. C-S-H gel is the main cementing compound during the curing process. It has a layer structure and contains both free water and chemically bound water. Calcium hydroxide nucleates and grows within free capillary pore spaces. It takes up about 20–25 vol% of the cement paste^2^. The problems associated with conventional OWC are low tensile strength and low fracture toughness^3^. In order to fulfill the critical function of cementing in the oil industry, different cement formulations, and reinforcing techniques have been applied.

Over decades, various additives have been incorporated into cement matrix to improve the rheological and mechanical properties of cement, including the use of silica fume^4^, fly ash^5^, and natural fiber^6^,^7^. For example, the resistance to both nucleation and growth of cracks was enhanced through adding a hybrid combination of steel and polypropylene fibers^3^. Carbon nanotube (CNT) reinforced cement has also received a great attention. The addition of CNTs to cement matrix changes the reinforcing mechanism from macro to nano level^8^. CNTs helped decrease electrical resistivity and increase the compressive sensitivity and Young’s moduli of cement^9^. CNTs also improved the durability properties of cement-based composites through decreasing the water permeability coefficient of cement mortar^10^. However, the use of CNTs in cement leads to high cost and non-sustainable. The aggregation of CNTs in cement matrix also causes significant property variation.

Cellulose is a biodegradable, renewable “green” material, which constitutes the most abandon renewable material available on the earth with approximately 7.5 billion tons produced annually through the condensation polymerization of glucose^11^. Cellulose is composed of β-1,4-linked D-glucopyranose (C_6_H_11_O_5_) units and the degree of polymerization (DOP) of native cellulose depended on the cellulose source material, such as plants, bacteria, tunicates and algae^12^. Cellulose fibers are widely used in composites to improve their mechanical properties^13^. With current advances in bio-based nanotechnology, cellulose nanoparticles (CNPs) have been extracted from various cellulose resources. CNPs offer some unique properties including high Young’s modulus, large aspect ratio, easy modification, and functionalization. CNPs have been previously tried as a reinforcing agent in a cementitious matrix. Mònica Ardanuy *et al*. reported the use of nanofibrillated cellulose (NFC) in the cement mortar composites^14^. Both sisal fiber and NFC reinforced cement mortar composites demonstrated improved flexural properties. In addition, the effect of cellulose nanocrystals (CNCs) on the physical properties of regular construction cement was investigated^15^. Water diffusion rate in high-density Calcium silicate hydrate was increased by CNCs and the maximum composite flexural strength was obtained with the addition of 0.2 vol.% of CNCs. It was also shown that the flexural strength of CNCs (0.2 vol. %) modified cement was increased by about 20–30%^15^. CNCs also promoted the degree of hydration of cement and improved the rheology properties of the slurry simultaneously. Cellulose nanofibers (CNFs) are another type of CNPs, which also exhibit great advantages when being used in composites as a reinforcing agent to improve mechanical and thermal properties. CNFs are composed of crystalline regions and amorphous regions. The Young’s modulus of crystalline region can reach up to aout 138 GPa in the longitudinal direction^16^. Furthermore, CNFs have a quite low coefficient of thermal expansion^17^ and large aspect ratio with length ranging from 500 to 2000 nm and diameter between 4 and 20 nm^18^. Bilodeau and Bousfield used CNFs to improve mechanical properties of particleboard and the fracture toughness was doubled when 20 wt.% of CNFs were added^19^.

OWC forms a pumpable slurry to seal the gap between casing and wellbore with excessive water bleeding. The rheology properties of OWC slurry have a tremendous effect on the workability, pumpability and final mechanical properties of OWC. Dousti discovered that viscosity and shear strain were changed significantly when CNCs were added into the cement slurry^20^. Flow tests showed that adding CNCs allowed cement slurries to withstand the low temperature regime^20^. Various factors (such as water to OWC ratios, additives, and OWC composition) influence the rheology of OWC slurry. Therefore, the rheological properties of OWC slurries need to be more accurately characterized^21^. Due to the shear thinning or thixotropic behavior of cement slurry, the Newtonian model is unsuitable to simulate the flow behavior of CNF/OWC cement colloids. Among the published work^22^, different rheological models have been used to aid the data analysis (e.g., determining the shear stress at zero shear rate)^23^,^24^. The Bingham plastic model is extensively used to determine the yield point and plastic viscosity of drilling fluids and cement slurries^25^. Like the Bingham plastic model, the Herschel-Bulkley model added the flow-behavior index, which is used to distinguish the types of fluids or slurries: pseudoplastic fluids (n < 1), Newtonian fluids (n = 1) and dilatant fluids (n > 1). Atzeni *et al*. obtained the optimum results with the Herschel-Bulkley model^26^. The Vocadlo model overcomes the shortcomings of other methods, which can be used to determine the actual yield stress rather than the property near the interface or wall^27^. The Vom Berg model has three adjustable parameters to fit the rheological behavior of cement slurries. Lapasin *et al*. modeled flow behavior of fresh cement paste successfully with this model^28^. No work has been reported applying these different models to the flow behavior of the CNF-OWC system.

The objective of this research was to test hypothesis that well-tuned CNFs can be used to improve flow rheology, curing, and strength properties of OWC through altering the cement’s shear thinning, hydration rate, and ability to bridge internal microcracks under stressing. Figure 1 outlines a general study scheme. Using thermal, diffraction and FTIR analyses, we demonstrated calcium hydroxide content, crystal structure and hydration products of hydrated OWC as influenced by the addition of CNFs. Static strength measurement coupled with morphological and elemental analysis showed OWC strength development during curing and CNF-reinforcing mechanism for the composite.

## Results

### Rheological Properties of CNF-OWC Slurry

Measured shear stress-shear rate curves and model fitting results are shown in Supplementary Figure S1 and Table S1, respectively. Obviously, the shear stresses increased with the increase of the shear rate and loading level of CNFs in the OWC matrix. With the addition of 0 (control), 0.04, 0.12, 0.20, 0.28 wt% of CNFs in the OWC matrix, the corresponding shear stresses were about 122, 194, 231, 257 and 290 Pa at the shear rate of 1000 s^−1^, respectively. The predicted values of yield stress and plastic viscosity follow the same trend. Using the predicted values from the Herschel-Bulkley model as an example, the yield stress of the reference slurry (no CNFs added) at the zero shear rate (τ_0_) was 19.19 Pa. τ_0_ gradually increased to 39.3 Pa when the ratio of CNF/OWC was 0.28 wt%. The plastic viscosity also increased from 0.109 to 0.271 Pa·s when the fraction of CNFs in OWC matrix increased from 0 to 0.28 wt%.

The slope of measured shear stress and shear rate curves of the CNF-OWC slurry decreased with the increase of shear rate, which reflected that OWC slurry is a type of shear-thinning fluid. The linear Bingham-Plastic model (Supplementary Figure S1a) is not very accurate in simulating shear rate-strain stress curve of the CNF/OWC slurry (R^2^ = 0.907–0.942). Shear stress was overestimated at low shear rate region and underestimated at high shear rate region with the Bingham-Plastic model. The Bingham-Plastic model is a two parameter (τ_0_ and μ_p_) model, which is still widely used in the oil industry. The Herschel-Bulkley model (Supplementary Figure S1b) incorporates the Bingham plastic model and the power law model together^29^. This three parameter model predicts the rheological behavior of OWC slurry better since the coefficient of determination (R^2^) ranges from 0.987 to 0.996 (Supplementary Table S1). The predicted power index n of the Herschel−Bulkley model, an indicator to evaluate the shear thinning behavior of OWC slurry^30^, decreased from 0.591 to 0.516 when more CNFs were added into the OWC matrix. This result indicates that OWC slurry with high ratios of CNFs exhibited a larger shear thinning effect. The Vocadlo model (Supplementary Figure S1c) also is suitable to fit the flow behavior of CNF/OWC slurry and the (R^2^ = 0.995). The best fitting results were obtained with the Vom Berg model (Supplementary Figure S1d) with the highest R^2^ value of 0.999. As summarized in Supplementary Table S1, the Vom-Berg model exhibits increased accuracy with the increased loading level of CNFs in OWC slurry, whereas Bingham-Plastic model shows an opposite trend.

A comparison of the predicted value of yield stress from the four rheology models is shown in Fig. 2. Obviously, the value of τ_0_ predicted by the Vocadlo model is the smallest in comparison with these from the other three models. The largest value was obtained from the Vom-Berg model. In addition, the standard error also increased sharply from 1.203 Pa to 14.860 Pa when using the Vocadlo model to fit the flow behavior with increased CNF levels into composites. One potential explanation is that the Vocadlo model has a higher accuracy in fitting data at high shear rate region. On the other hand, the Vom-Berg model provides more accurate fitting at low shear rate region.

### Degree of Hydration (DOH) of Hydrated CNF-OWC Composites

The TGA curves (140 °C–1000 °C) of various CNF modified type H OWC formulations are shown in Fig. 3a. Portlandite concentration and the corresponding DOH values are plotted in Fig. 3b. The temperature level 140 °C was chosen as the starting temperature and the weight at this temperature was used as the base (100%). The weight loss from ambient temperature to 140 °C is considered as the removal of physically bonded water (PBW) or free water, and the weight loss between 140 °C and 1000 °C corresponds to the evaporation of chemically bonded water (CBW)^31^. The weight loss in the low temperature zone (<400 °C) corresponded to the decomposition of complex hydrated silicate or aluminate compound. In addition, all hydrated OWC samples exhibited a weight loss in the temperature range 440 °C to 520 °C, which can be ascribed by the decomposition of portlandite^32^. The weight loss associated with the decomposition of calcium carbonate took place at higher temperature regions (600 °C–780 °C). As shown in Fig. 3a, the final weight loss of hydrated OWC samples decreased from 90.4% to 86.2% when the weight ratio of CNF/OWC increased from 0 to 2.66%. It is apparent that the addition of CNFs in the OWC promoted the reaction between water and OWC. The DOH data of hydrated OWC samples with various concentrations of CNFs was calculated using Supplementary Equation S5, and the corresponding results are shown in Fig. 3b. The values of DOH increased from 41.6% to 60.1% with the increase of CNF concentration in the OWC matrix (Table 1).

Apparently, DOH was improved by the addition of CNFs. Since Ca(OH)_2_ is a product of chemical equation of C_3_S and C_2_S, portlandite is considered as an indicator of the hydration process^33^. The Mounanga’s method (Supplementary Equation S6) is used to determine the portlandite concentration in the hydrated OWC samples. Portlandite concentration followed a similar increasing trend, which is consistent with the trend of DOH. The concentration of calcium hydroxide increased from 16.4 to 18.4 when the loading of CNFs was increased from 0 to 0.28%. The portlandite concentration also indicated that the DOH value was promoted with the addition of CNFs in the OWC matrix. Alite (C_3_S) and belite (C_2_S) took up at least 55% of the anhydrate type H well OWC. During the hydration process, C_3_S and C_2_S formed calcium silicate hydrate (3CaO·2SiO_2_·4H_2_O or C-S-H) gel and portlandite as shown in the following chemical reactions.


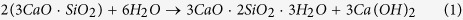



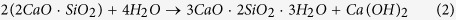


C-S-H gel formed high density shell and wrapped up these anhydrate OWC particles. It inhabited further hydration reactions. However, CNFs provided a pathway to transport water into the core. The surface of CNFs had many hydroxyl groups, which helped promote water sorption.

DSC curves of CNF-OWC composites are shown in Fig. 3c and the corresponding enthalpy changes are summarized in Fig. 3d. Two major peaks and three smaller peaks are seen in the DSC curves. The first endothermic peak at 120 °C was due to the water loss from calcium-silicate-hydrate gel. Since there were two types of water molecules (free water and chemically bond water) in the CNF modified OWC, another smaller sub-peak observed at 96 °C was due to water evaporation. The endothermic peak at 140 °C and the shoulder at 405 °C were due to the iron-substituted ettringite and Fe_2_O_3_ solid solution, which are the reaction product of tetracalcium alumino-ferrite (4CaO·Al_2_O_3_·Fe_2_O_3_). The second major peak at 526 °C is attributed to the decomposition of calcium hydroxide. The enthalpy change (Fig. 3d) followed the same trend as calcium hydroxide concentration measurement in Fig. 3b. The enthalpy of decomposition of Ca(OH)_2_ increased from 45.5 J/g to 72.9 J/g with increased CNF/OWC weight ratio (Table 1). The results are in a good agreement with those provided by TGA data.

X-ray diffraction data of the CNF-OWC composite shown in Supplementary Figure S2 further showed increased peak intensity due to calcium hydroxide (2θ = 18° in Supplementary Figure S2c) with the addition of CNFs into the OWC matrix. The increased formation of potlandite indicated higher degree of hydration. The increased values (Table 1 and Supplementary Figure S2d) of the index of degree of hydration, IDOH, representing a ratio of integrated diffraction peak area of portlandite to that of alite also showed an increase of OWC hydration with increased weight ratio of CNF to OWC, consistent with the quantitative characterization by the thermal analysis.

### Morphology and Mechanical Properties of Hydrated CNF-OWC Composites

Typical FTIR spectra of the CNF-OWC composite cured for 28 days as shown in Supplementary Figure S3 provide a positive evidence for the existence of CNFs in the OWC matrix. The SEM images of actual CNFs and hydrated composite paste are shown in Fig. 4a–d, and the element composition analysis data for selected composite regions are illustrated in Fig. 4e,f 34.

CNFs had a large aspect ratio with an average diameter of 20 nm and length over several microns (Fig. 4a). Figure 4b shows a typical “pull-out mechanism” of the low weight fraction of CNFs (0.04 wt%) in OWC composite. CNFs distributed rather homogeneously in the OWC matrix at the low CNF loading levels. However, when the loading of CNFs was increased, agglomeration phenomenon appeared (Fig. 4d). CNF aggregation certainly influenced the mechanical properties of the hydrated OWC composites. Figure 4c presents the fracture surface of the CNF-OWC composites. CNFs helped bridge different parts of OWC together, forming micro-bridges. The FTIR and element composition analyses of hydrated OWC composite confirmed the existence of CNFs, since the peak intensity of carbon exhibits an obvious difference in Fig. 4e,f. The peak of platinum was due to the Pt coating on test samples. The elements shown in the EDS spectra are similar to those reported in the previous published papers^35^.

Average flexural strength as a function of CNF loading levels measured at three different curing stages (i.e., 10, 20, and 30 days) is shown in Fig. 5. The flexural strength increased with the incorporation the CNFs at the low loading levels. The strength values peaked at 13.93 MPa corresponding to the CNF loading level of 0.04 wt%. The composite strength finally decreased with further increase of CNFs. Compared with the strength of OWC without CNFs, the flexural strength of the CNF-OWC composites at the CNF loading level of 0.04 wt% increased about 23.9%, indicating a significant reinforcing effect of CNFs.

## Discussion

The initial increase of the composite flexural strength was attributed to several reasons. According to the law of mixtures (*σ*_*c*_* = σ*_*m*_ *V*_*m*_* + σ*_*f*_ *V*_*f*_ ), the strength of fiber reinforced composites *σ*_*c*_ is associated with the strength of the matrix (*σ*_*m*_), the strength of fiber (σ_f_) and their respectively volume fractions (*V*_*m*_, and *V*_*f*_)^36^. The strength of CNFs is estimated at about 2–6 GPa^37^, which is much higher than the strength of hydrated OWC. Thus, when a small amount of CNFs was added to OWC matrix, the strength of the CNF-OWC composites increased to some extent due to strengthening of CNFs.

**Figure 1:**
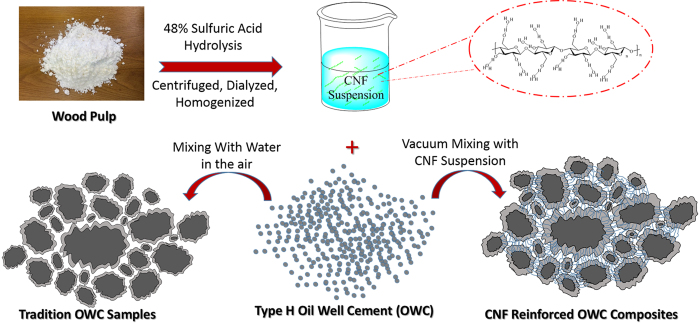
Schematic of the preparation method of cellulose nanofiber reinforced OWC composites.

**Figure 2:**
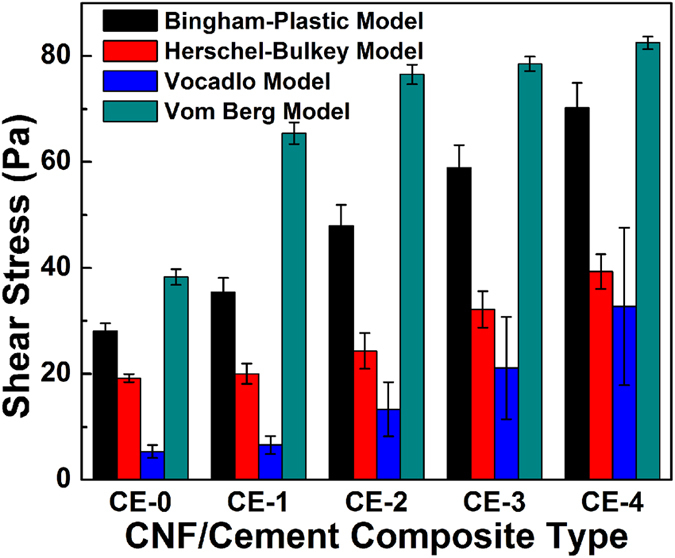
Predicted yield stresses of CNF-OWC composite slurries from different rheology models.

**Figure 3:**
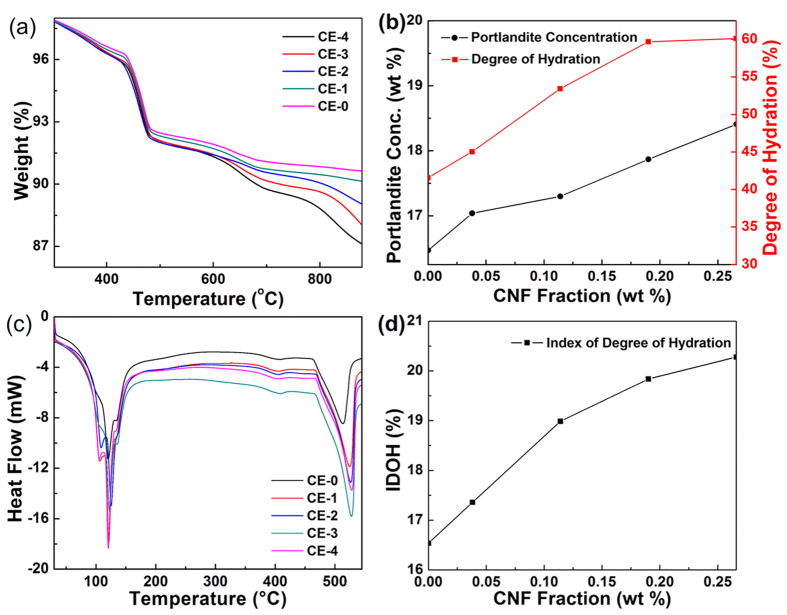
Degree of hydration properties of CNF-OWC composites. (**a**) TGA curves, (**b**) calculated degree of hydration and Ca(OH)_2_ concentration as a function of CNF loadings, (**c**). DSC curves, and calculated enthalpy change as a function of CNF loading (**d**).

**Figure 4:**
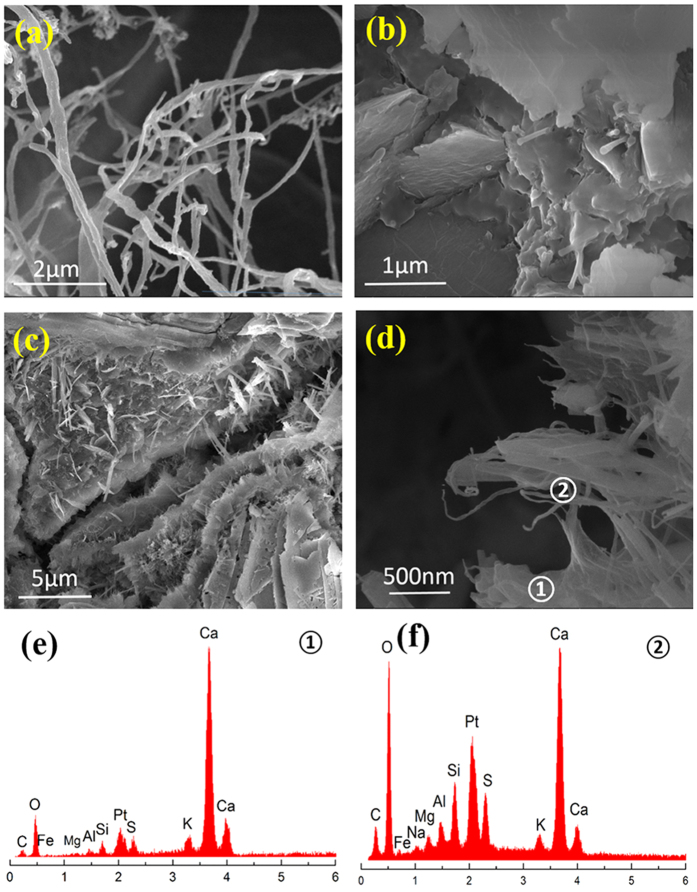
Micrographs of CNFs and CNF-OWC composites and related composition analysis: (**a**) CNFs, (**b**) CNF-OWC composite with 0.04 wt% CNF loading, (**c**) CNF-OWC composite with 0.28 wt% CNF loading, (**d**) CNF aggregation in OWC matrix, (**e**) EDS spectra of area 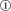 in (**d**, **f**) EDS spectra of area 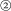 in (**d**).

**Figure 5:**
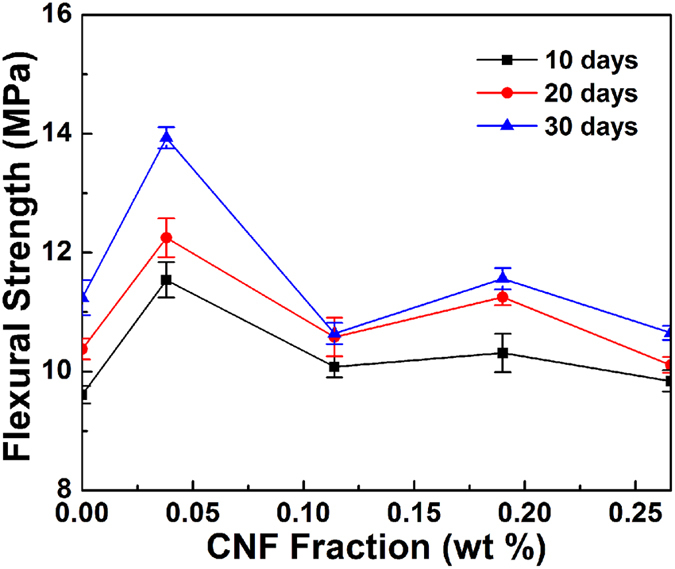
Flexural strength of CNF-OWC composites as a function of CNF loading at three different curing times.

Many factors influenced the mechanical properties of CNF-OWC composites. CNFs, with typical thermal stability, surface functional groups, and crystalline structure properties shown in Supplementary Figures S4, S5, and F6, respectively, have large surface area (140–160 m^2^/g^−1^ for free dried CNFs) and high surface free energy (56.72 mJ/m^2^)^38^, which can enhance the degree of hydration of the hydrated OWC paste and finally improve the strength of OWC. The surface of CNFs had many hydroxyl groups (Fig. 1), which helped enhance the hydration process of CNF-OWC composites. As a result, the area of hydrated OWC was increased with the use of CNFs in comparison with control. The diameter of CNFs is about 4–20 nm and its length ranged from 500 to 2000 nm. CNFs had a quite high aspect ratio (30–300)^39^. The large aspect ratio of CNFs not only can divert and block micro cracks, but it can also prevent crack propagation through the “bridge effect”. As shown in Figs 1 and 4b and. CNFs helped link different parts of OWC together. Finally, it is unavoidable that OWC had various sizes of pores, which are detrimental to the mechanical properties of OWC paste^40^. As a typical nanomaterial, CNFs played a role in decreasing the porosity of OWC, which can improve the mechanical properties of interfacial transition zone in the OWC paste. The CNF-OWC composites at the 0.04 wt% CNF loading level still exhibited the best flexural strength value among all formulations even after aging in the air for 382 days, indicating stable material composition.

The final decrease of the flexural strength of OWC composite was ascribed by the insufficient dispersion of CNFs in the OWC paste. As clearly seen in Fig. 4c, the aggregation of CNFs formed weak zone in the OWC. When the external force was applied on the OWC sample, micro cracks were formed at these weak zones first. Then stress concentration increased the crack propagation rate and finally caused the failure of OWC composite^41^.

## Conclusion

Our data suggested that the yield stress of fresh CNF/OWC slurries increased with the increased loading of CNFs in OWC composites. The Vom Berg model demonstrated the best fitting results of the flow rheology behavior. Thermal analysis demonstrated the increased trend of DOH and the rising concentration of calcium hydroxide during the hydration process with the addition of CNFs. The flexural strength of hydrated CNF-OWC samples peaked at 13.93 MPa with the addition of 0.04 wt% CNFs in the OWC matrix. The flexural strength increased up to 20.7% in comparison with that of the control samples. The increase of mechanical properties was attributed to the increased DOH and the binding effect of homogeneously distributed CNFs in the OWC matrix as demonstrated through SEM-EDS analysis on the surface of the hydrated OWC. However, flexural strength gradually decreased with the excessive addition of CNFs due to the aggregation of CNFs in the OWC matrix (i.e., forming clusters of CNFs). Overall, the CNF-reinforced OWC exhibits better mechanical and rheological properties compared with the original OWC. CNFs, as a high strength, inexpensive, renewable and little health/safety risk material, are demonstrated to be an excellent, sustainable reinforcing nanomaterial to improve the performance of oil well cement.

## Experiment Section

### Materials

Bleached wood pulp (W-50 grade) was provided by Nippon Paper Chemicals Co., Ltd. (Tokyo, Japan). Sulfuric acid (98%, EMD Millipore Corporation, Chicago, IL, USA) was diluted to a concentration of 48 wt % before use. Type H well cement (Halliburton Energy Services, Houston, TX, USA). ASTM Type II deionized water with a resistivity of 10 MΩ cm was purchased from VWR International LLC (Radnor, PA, USA). Regenerated cellulose dialysis tubing was purchased from Fisher Scientific (Pittsburgh, PA, USA).

### Preparation of Cellulose Nanofibers

48 wt% sulfuric acid (fiber-to-acid weight ratio of 1 to 10) was used to hydrolyze bleached wood pulp at 45 °C for about 1 h. Deionized water was then added to dilute the acid and pulp mixture to 10 folds to stop the reaction. The material was centrifuged to remove most sulfuric acid in the diluted suspension. The residual acid was further removed through dialyzing in 3 to 5 days using the regenerated cellulose dialysis tube. After neutral pH was reached, the obtained aqueous suspension was passed through a high-pressure homogenizer (Microfluidizer Processor M-110P, Microfluidics Corp., Newton, MA, U.S.A.) five times under an operating pressure of 200 MPa.

### Preparation of CNF-OWC Composites

Different weight ratios of CNFs and class H OWC were mixed (Table 1). The temperature was maintained at 23 °C ± 1 °C during the mixing process. The samples were prepared according to the API Specification 10A with a water to OWC ratio of 0.38. A vacuum mixer (AX-200, Tianjin Share Import and Export Co., Tianjin, China) was used in the mixing procedure in order to eliminate any bubbles in the CNF-OWC slurry for subsequent slurry rheology and cured composite analysis. For preparing cured composite samples, freshly mixed CNF-OWC slurry was poured into a metal prism mold (50 mm × 50 mm × 250 mm) to prepare test specimens. The samples were cured at room temperature and were demolded after 24 hours curing.

### Characterization Method

More details on characterization methods can be found at Supplementary Information section. In brief, the rheology properties of fresh CNF-OWC slurry were characterized a stress controlled rheometer (AR2000ex, TA Instruments Inc., New Castle, DE, USA) at room temperature with a shear rate range from 1 s^−1^ to 1000 s^−1^. Four different models (Supplementary Equations S1–S4) were used to fit the recorded shear stress- shear strain curves. Degree of hydration (DOH) of CNF-OWC composites was performed using a thermo-gravimetric analyzer TGA (TA Q50 Instruments Inc., New Castle, DE, USA) at the temperature range of 30–1000 °C^42^. The Differential Scanning Calorimetry (DSC) measurement was performed using a TA Q2000 calorimeter (TA Instruments Inc., New Castle, DE, USA) between 30–550 °C. The portlandite content in the hydrated composite was calculated by using Supplementary Equations S5 and S6.Wide-angle X-ray diffraction (WXRD) patterns of CNF-OWC composites were obtained using a Bruker/Siemens D5000X-ray automated powder X-ray diffractometer (Billerica, MA, USA) in the angular range of 5° to 60°. FTIR spectra of hydrated CNF-OWC composite were recorded through a Bruker Fourier Transform Infrared Spectrometry (FTIR) analyzer (Billerica, MA, USA) over the range of 4000-500 cm^−1^. The fracture surface of CNF-OWC samples was observed by a Quanta™ 3D DualBeam™ FESEM (FEI Company, Hillsboro, OR, USA) at 10 kV. The elemental composition was determined through an integrated energy dispersive electron microprobe system (EDS system) with an accelerating voltage of 20 kV.Flexural strength of hydrated CNF-OWC composites sample were measured using an Instron 5582 universal testing machine (Instron Company, Norwood, MA, USA) equipped with a 100 KN load cell.

## Additional Information

**How to cite this article**: Sun, X. *et al*. Cellulose Nanofibers as a Modifier for Rheology, Curing and Mechanical Performance of Oil Well Cement. *Sci. Rep.* **6**, 31654; doi: 10.1038/srep31654 (2016).

## Supplementary Material

Supplementary Information

## Figures and Tables

**Table 1:** Formulation and Summarized Properties of CNF-OWC composites.

Sample No.	Formulation (PPH)			Characterization Results			
	OWC	Water	CNFs	DOH (%)	Enthalpy (J/g)	Ca (OH)_2_ (%)	IDOH
CE-0	100	38	0	41.57	45.5	16.47	0.1654
CE-1	100	38	0.04	45.04	58.7	17.04	0.1736
CE-2	100	38	0.12	53.43	62.6	17.3	0.1899
CE-3	100	38	0.20	59.7	67.3	17.87	0.1984
CE-4	100	38	0.28	60.13	72.9	18.41	0.2028

Note: OWC: Oil well cement, CNFs: Cellulose nanofibers, and PPH: Parts per hundred, DOH: Degree of Hydration, and IDOH: Index of degree of hydration.
