# Supplementary material for: Cellulose Nanofibers as a Modifier for Rheology, Curing and Mechanical Performance of Oil Well Cement
Source: Sci Rep. 2016 Aug 16;6:31654. doi: 10.1038/srep31654 (PMC4985826; doi:10.1038/srep31654)
Supplement: Supplementary Information [file srep31654-s1.doc]

**Supplementary Information**

**Cellulose Nanofibers as a Modifier for Rheology, Curing and Mechanical Performance of Oil Well Cement**

Xiuxuan Sun^1^, Qinglin Wu^1^,*, Sunyoung Lee^2^, Yan Qing^3^ and Yiqiang Wu^3^,*

^1^School of Renewable Natural Resources, Louisiana State University AgCenter, Baton Rouge, Louisiana 70803, United States. ^2^Department of Forest Products, Korea Forest Research Institute, Seoul, 130-712, Korea. ^3^College of Materials Science and Engineering, Central South University of Forestry and Technology, Changsha, 410004, China.

Correspondence and requests for materials should be addressed to

Q.W. (E-mail: [qwu@agcenter.lsu.edu](mailto:qwu@agcenter.lsu.edu)) and Y.W. (E-mail: [wuyiqiang@csuft.edu.cn](mailto:wuyiqiang@csuft.edu.cn)).

**Characterization of** **CNF-OWC Composites**

**Slurry Rheology.** A stress controlled rheometer (AR2000ex, TA Instruments Inc., New Castle, DE, USA) was used to characterize the rheology properties of fresh CNF-OWC slurry. All measurements were performed using a cone and plate geometry (Angle: 2^o^, Diameter: 60 mm) at room temperature with a shear rate range from 1 s^-1^ to 1000 s^-1^. Shear stresses as a function of shear rate for various formulations were collected and analyzed. Four different models were used to fit the recorded shear stress- shear strain curves, including:

Bingham-Plastic Model:


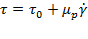
 (S1)

Herschel-Bulkley model:


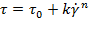
 (S2)

Vocadlo model:


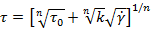
 (S3)

Vom Berg model:


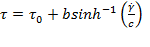
 (S4)

In the equations S1-S4, τ denotes shear stress (Pa),
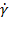
 is the shear rate (s^-1^), τ_0_ is yield stress (Pa) at zero shear strain rate, μ_p_ is the plastic viscosity of the OWC slurry (Pa·s^-1^) and *k, n, b, c* are the model parameters.

**Composite Property.** Degree of hydration (DOH) of CNF-OWC composites was studied using a Q50 thermo-gravimetric analyzer (TGA, TA Instruments Inc., New Castle, DE, USA) in nitrogen.[^1^](#_ENREF_31) Approximately 50 mg of hydrated composite samples were placed into a platinum pan and heated at a uniform heating rate of 10 ^o^C/min. In order to remove any free water in the hydrated OWC sample, the sample was heated from room temperature to 140 ^o^C and the temperature was maintained for 15 minutes. Then the temperature was increased from 140 ^o^C to 1000 ^o^C. The weight loss between 140 ^o^C and 1000 ^o^C was considered as the weight of chemically bound water (CBW) and the DOH of hydrated composite sample was calculated using Equation S5:


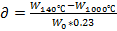
 (S5)

where
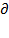
 is the degree of hydration, *W_140_^o^_C_* and *W_1000_^o^_C_* are the weight of samples at 140^o^C and 1000 ^o^C, respectively; *W_0_* is the initial weight of the sample, and the factor 0.23 is the weight of non-evaporable water content per unit gram of unhydrated composite sample.[^2^](#_ENREF_41)

The hydration characteristics of CNF-OWC composites were also measured by calculating the calcium hydroxyde (portlandite) content in the hydrated composite. Since the decomposition of Ca(OH)_2_ occurred in the region 400°C to 600 °C, Mounanga’s method (Equation S6)was used.[**^3^**](#_ENREF_42)**,**[**^4^**](#_ENREF_32)


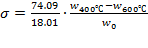
 (S6)

where σ is the portlandite content of the hydrated composite, *W_400_^o^_C_* and *W_600_^o^_C_* are the weight of samples at 400^o^C and 600 ^o^C, respectively; *W_0_* is the initial weight of the sample, and the factor (74.09/18.01) is the molar mass ratio of portlandite and water.

Differential Scanning Calorimetry (DSC) measurement was performed using a TA Q2000 calorimeter (TA Instruments Inc., New Castle, DE, USA) in nitrogen. Each hydrated sample of about 10 mg was sealed in an aluminum pan and was heated from 30 ^o^C to 550 ^o^C at a heating rate of 10 ^o^C/min. Heat flow rate as a function of temperature was collected for each sample. The enthalpy change related to the decomposition reaction was investigated by integrating the peak area of calcium hydroxide and the results.

Wide-angle X-ray diffraction (WXRD) patterns of CNF-OWC composites were obtained using a Bruker/Siemens D5000X-ray automated powder X-ray diffractometer (Billerica, MA, USA). The WXRD data were generated by the diffractometer with Cu-Ka radiation (λ = 1.54Å) at an accelerating voltage of 45 kV and a current of 40 mA. The diffracted intensity for each composite formulation was determined in the angular range of 5º to 60º at a step size of 0.026^o^.

A Bruker Fourier Transform Infrared Spectrometry (FTIR) analyzer (Billerica, MA, USA) was used to record FTIR spectra of hydrated CNF-OWC composite. All spectra over the range of 4000-500 cm^-1^ after 32 scans with a resolution of 4 cm^-1^ were acquired on a Zn/Se ATR crystal cell under transmittance mode.

The fracture surface of CNF-OWC samples was observed by a Quanta™ 3D DualBeam™ FESEM (FEI Company, Hillsboro, OR, USA) under 10 kV. Small pieces of hydrated CNF-OWC were mounted on standard SEM aluminum stand and the samples were coated with a thin layer of Pt. The elemental composition was determined through an integrated energy dispersive electron microprobe system (EDS system) with an accelerating voltage of 20kV.

Flexural strength of hydrated CNF-OWC composites sample were measured using an Instron 5582 universal testing machine (Instron Company, Norwood, MA, USA) equipped with a 100 KN load cell. The dimensions of the specimen were 50mm x 50mm x 250mm. The rectangular composite prisms were loaded in a three point loading mode at a crosshead speed of 0.5 mm/min with a span length of 200mm.

**Characterization Results of CNF-OWC Composites**

**Rheology Data.** Figure S1 shows experiment data and fitted shear stress-shear rate curves of CNF-OWC slurries with four different models (i.e., Bingham-Plastic model, Herschel-Bulkey model, Vocadlo model, and Vom Berg model). Table S1 summarizes parameters from the four rheological models at different CNF loading levels.


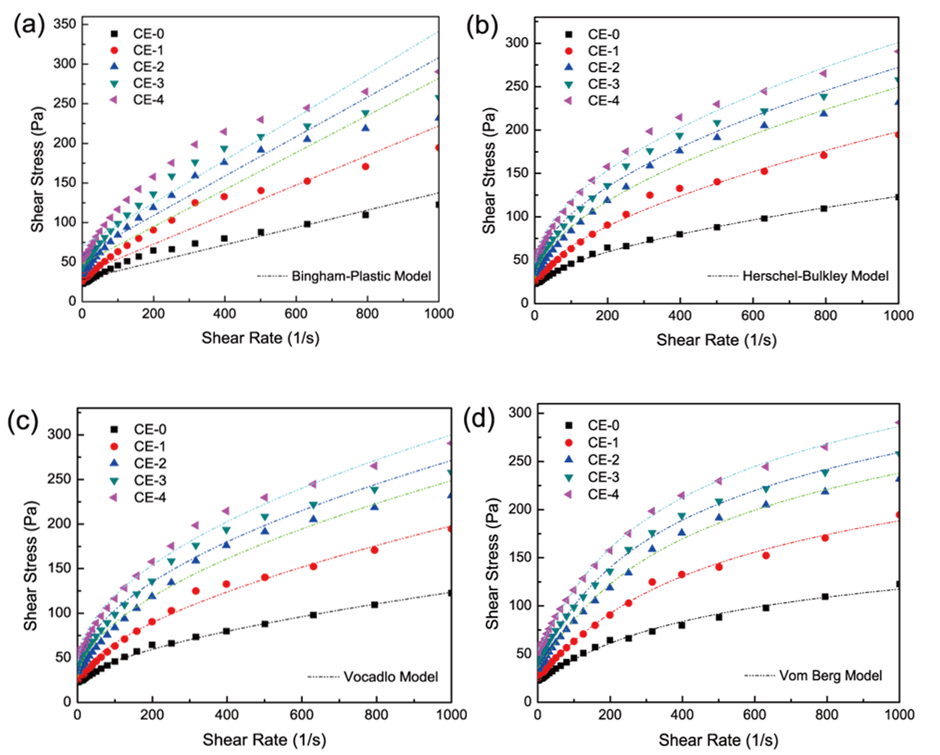


**Figure S1.** Experiment data and fitted shear stress-shear rate curves of CNF-OWC slurries from (a) Bingham-Plastic model, (b) Herschel-Bulkey model, (c) Vocadlo model, and (d) Vom Berg model.

**Table S1. Rheological model parameters for CNF-OWC slurries**

| Rheological models | | CNFs (wt % based on OWC weight) | | | | |
| --- | --- | --- | --- | --- | --- | --- |
|  |  | 0 | 0.04 | 0.12 | 0.20 | 0.28 |
| Bingham-Plastic  Model | τ_0_ | 28.103 | 35.447 | 47.957 | 58.951 | 70.264 |
|  | μ_p_ | 0.109 | 0.187 | 0.234 | 0.249 | 0.271 |
|  | R^2^ | 0.942 | 0.934 | 0.911 | 0.908 | 0.907 |
| Herschel-Bulkey  Model | τ_0_ | 19.191 | 20.021 | 24.333 | 32.163 | 39.304 |
|  | k | 1.756 | 3.053 | 5.219 | 6.160 | 7.411 |
|  | n | 0.591 | 0.589 | 0.545 | 0.530 | 0.516 |
|  | R^2^ | 0.996 | 0.991 | 0.987 | 0.989 | 0.992 |
| Vocadlo  Model | τ_0_ | 5.333 | 6.566 | 13.293 | 21.099 | 32.709 |
|  | k | 0.968 | 1.709 | 3.789 | 5.011 | 7.027 |
|  | n | 0.762 | 0.808 | 0.912 | 0.945 | 0.979 |
|  | R^2^ | 0.995 | 0.991 | 0.986 | 0.988 | 0.992 |
| Vom-Berg  Model | τ_0_ | 38.271 | 65.451 | 76.548 | 78.554 | 82.528 |
|  | b | 0.006 | 0.006 | 0.007 | 0.008 | 0.008 |
|  | c | 23.133 | 26.792 | 35.142 | 44.477 | 53.693 |
|  | R^2^ | 0.996 | 0.997 | 0.998 | 0.999 | 0.999 |

**XRD Data.** The effect of CNFs on the hydration characteristics of the OWC was further examined by the XRD technique using hydrated composites (28 days) with different CNF loading levels (Figure S2). Typical diffraction peaks of type H oil well OWC were identified and are indexed in Figure S2a. All the major components–of the material, including alite (α), belite (β), ettringite (ε) and ferrite (φ) and their reaction product -portlandite (ρ) and calcite (ψ) were found in the diffraction patterns of the CNF-OWC composites (Figure S2b). No obvious difference in the diffraction patterns of CNF modified OWC was observed since only a quite small amount of CNFs was added into the OWC matrix. It also revealed that no alteration occurred in the phases present. The formation of calcite was due to the reaction of portlandite or C-S-H gel with carbon dioxide in the atmosphere. Some peaks of the components of hydrated OWC paste were overlapped. For example, the peaks of alite, belite, portlandite and calcite were overlapped at 34^o^. The noticeable features observed in the X-ray diffractogram of the CNF-OWC composite were that the diffraction peaks due to calcium hydroxide (2θ=18^o^) showed increased intensities (Figure S2c) with addition CNFs into OWC matrix. The increased formation of potlandite indicated higher degree of hydration. These results are entirely consistent with the quantitative characterization estimated by thermal analysis (TGA and DSC method), when considering the peak intensity as a measure of the quantity of calcium hydroxide present in the CNF modified well OWC. As mentioned above, portlandite was produced because of the hydration of alite. The diffraction peak of alite was observed at 29.5^o^. Thus, the index of degree of hydration, IDOH, can be obtained through calculating the ratio of integrated diffraction peaks of portlandite to alite from XRD analysis.^5^ The calculated results are presented in Figure S2d and Table 1. The integrated peak area ratio of ρ to α reached to 0.2028 from 0.1654. It is obvious that increasing the weight ratio of CNF/OWC resulted in a slight increase of the OWC hydration.


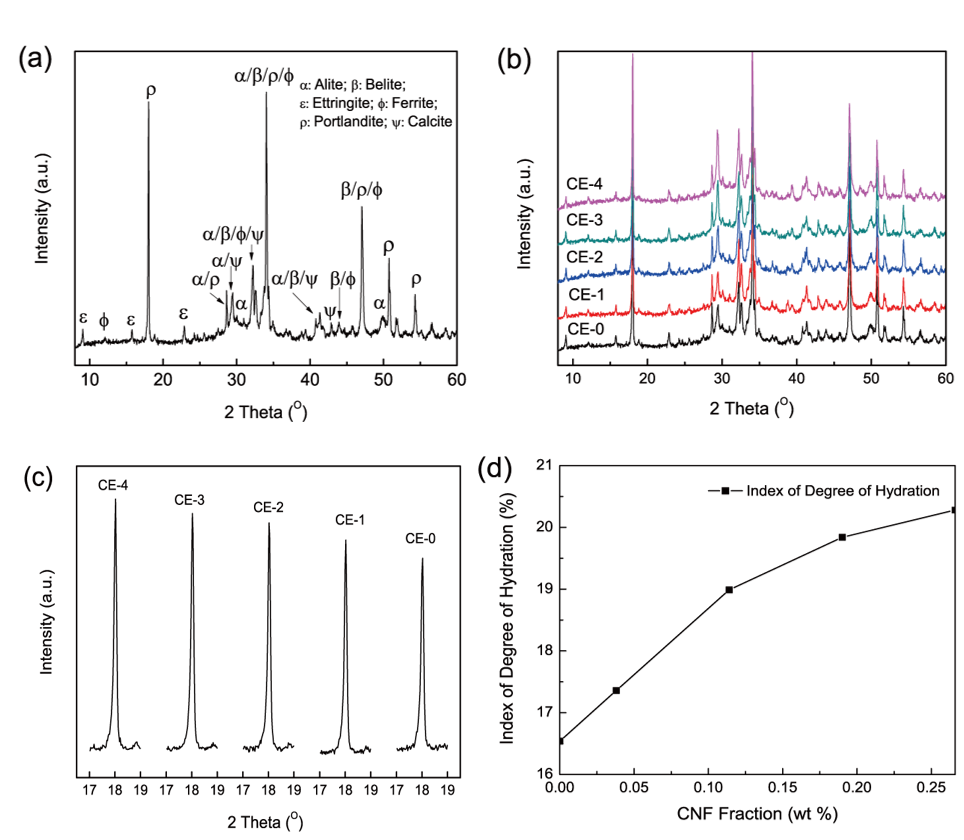


**Figure S2.** XRD analysis and predicted Index of Degree of Hydration (IDOH) of CNF-OWC composites: (a) Indexed XRD pattern, (b) XRD patterns, (c) peak intensity and (d) IDOH as a function of CNF loading.

**FTIR Data.** Typical FTIR spectra of the composite cured for 28 days to demonstrate hydrated kinetics are shown in Figure S3. FTIR data of the hydrated OWC specimens provided a positive evidence for the existence of CNFs in the OWC matrix. The peak intensities increased gradually with increased CNF levels. Specifically, the peak at 875 cm^-1^ was not observed in the FTIR spectra of the control sample CE-0 (i.e. no CNFs added). However, when the weight ratio of CNF/OWC increased to 0.28%, the peak can be clearly seen at the same position (Red circle marked area in Figure S3). The other main vibrational bands related to OH-groups of calcium hydroxide, symmetric stretching (ν_1_) and asymmetric stretching (ν_3_) of O-H vibrator of water, in-plane Si-O bending (ν_2_ SiO_4_^4-^) and asymmetric Si-O stretching (ν_3_ SiO_4_^4-^) were also identified. The small narrow band observed at 3635 cm^-1^ is attributed to stretching vibration of OH-groups of calcium hydroxide. The broad band appearing at 3405 cm^-1^ is assigned to ν_1_ and ν_3_ of O-H vibrator of water. Another broad band at 1645 cm^-1^ is due to ν_2_ deformation mode of H-O-H bond. Since the OWC slurry was cured in air, the reaction of carbon dioxide with calcium dioxide occurred. The sharp band at 1430 cm^-1^ (ν_3_ CO_3_^2-^) confirmed the occurrence of this phenomenon. The band at 1112 cm^-1^ and 953 cm^-1^ was due to the asymmetric Si-O stretching, which reveals the polymerization of SiO_4_^4-^ tetrahedral units present in the hydrated composite during the formation of calcium silicate hydrate gel. In addition, the shoulder at 820 cm^-1^ is ascribed to ν_2_ SiO_4_^4-^.


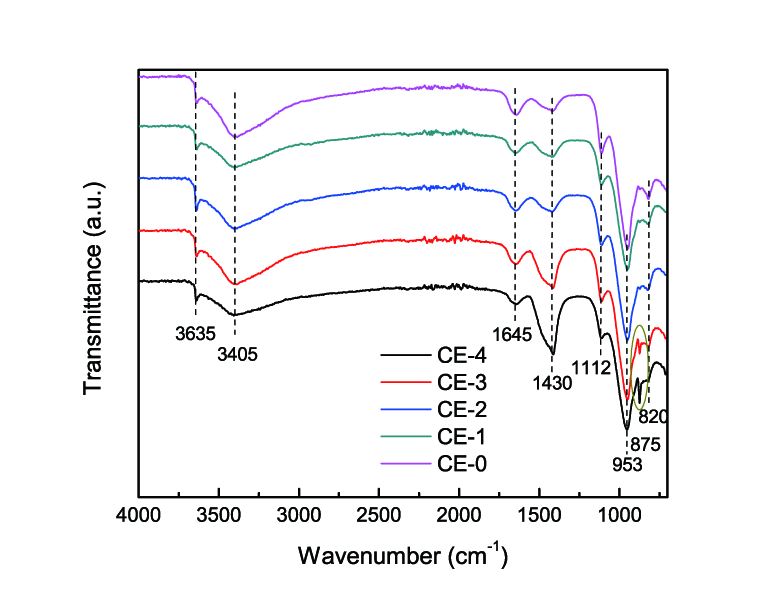


**Figure S3.** FT-IR transmittance spectra of hydrated OWC composites for different CNF-OWC formulations.

**Characterization Results of CNFs**

Figures S4, S5, and S6 show basic properties of CNFs used in the study.


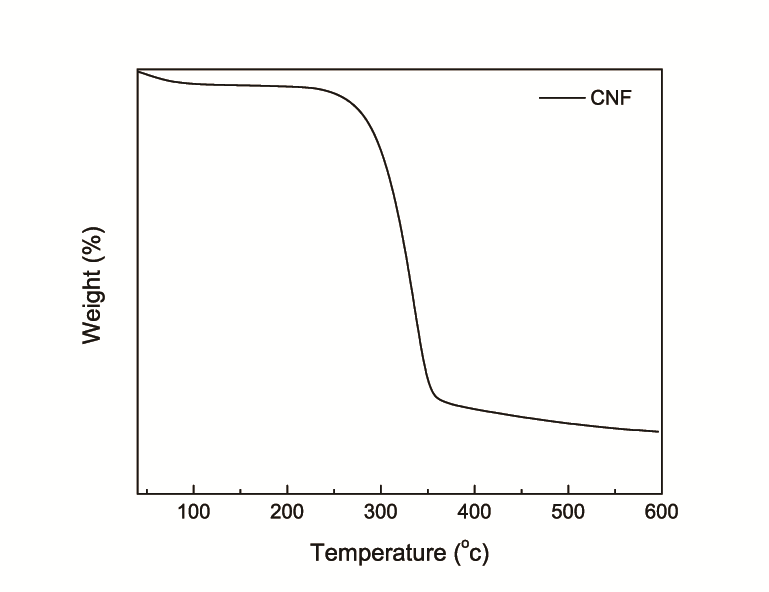


Figure S4. TGA curve of CNFs.

Figure S5. FT-IR transmittance spectra of CNFs.


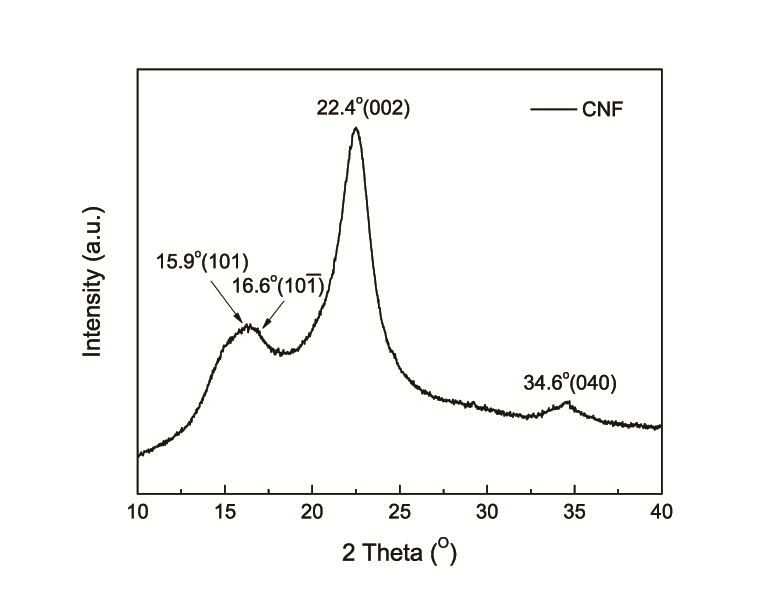


Figure S6. X-ray diffraction spectra of CNFs.

References:

1. Pane, I.; Hansen, W., Investigation of blended cement hydration by isothermal calorimetry and thermal analysis. *Cem Concr Res***,** ***35***, 1155-1164 (2005).

2. Cao, Y.; Zavaterri, P.; Youngblood, J.; Moon, R.; Weiss, J., The influence of cellulose nanocrystal additions on the performance of cement paste. *Cem Concr Compos***,** ***56***, 73-83 (2015).

3. Midgley, H. G., The determination of calcium hydroxide in set Portland cements. *Cem Concr Res***,** ***9***, 77-82 (1979).

4. Mounanga, P.; Khelidj, A.; Loukili, A.; Baroghel-Bouny, V., Predicting Ca(OH)2 content and chemical shrinkage of hydrating cement pastes using analytical approach. *Cem Concr Res***,** ***34***, 255-265 (2004).

5. Yousuf, M.; Mollah, A.; Lu, F.; Schennach, R.; Cocke, D. L., An X-Ray Diffraction, Fourier-Transform Infrared Spectroscopy, and Scanning Electron Microscopy/Energy-Dispersive Spectroscopic Investigation of the Effect of Sodium Lignosulfonate Superplasticizer on the Hydration of Portland Cement Type V. *Polym Plast Technol Eng***,** ***38***, 849-868 (1999).
